# An assessment of the effectiveness of an electronic wristband in improving routine immunization timeliness and reducing drop-out

**DOI:** 10.1093/pubmed/fdad134

**Published:** 2023-08-08

**Authors:** Sidney Sampson, Adebisi Adenipekun, Sunday Atobatele, Oluwafisayo Ayodeji, Oluomachukwu Omeje, Folake Oni

**Affiliations:** Sydani Group, Plot 1422, Independence Avenue, Central Business District, Abuja, Nigeria; Sydani Group, Plot 1422, Independence Avenue, Central Business District, Abuja, Nigeria; Sydani Group, Plot 1422, Independence Avenue, Central Business District, Abuja, Nigeria; Sydani Group, Plot 1422, Independence Avenue, Central Business District, Abuja, Nigeria; Sydani Group, Plot 1422, Independence Avenue, Central Business District, Abuja, Nigeria; Sydani Group, Plot 1422, Independence Avenue, Central Business District, Abuja, Nigeria

**Keywords:** Immunization, timeliness, reminder, dropout rates, caregivers, vaccine

## Abstract

**Background:**

To assess the effectiveness of a wristband for immunization alert (WIA) as a reminder device to caregivers to improve immunization timeliness and reduce drop-outs.

**Methods:**

Eight health facilities, selected from two local government areas in Kano state, Northwestern Nigeria, were clustered in a two-arm study involving an intervention group and a control group. Only the caregivers (757) from the intervention group received WIA as an immunization reminder device. Immunization timeliness data were then collected from the control and intervention groups for the period of intervention and analyzed using Microsoft Excel and IBM SPSS version 21.

**Results:**

A cohort analysis of caregivers who received WIA at their second visit showed an increase in immunization timeliness from 10% at the second visit to 86% at the third visit and maintained at 66% for the fifth visit. A difference-in-difference analysis of the effect of WIA on immunization timeliness from baseline to end-line in the control and intervention groups showed a positive 30% increase in immunization timeliness associated with the introduction of WIA.

**Interpretation:**

Given that immunization timeliness and drop-outs are reported issues of concern in Northwestern Nigeria, the use of the WIA device is a recommended intervention.

## Introduction

Immunization is a proven cost-effective public health strategy to prevent morbidity and mortality, especially in children and infants.^1–4^ However, there are many factors such as availability, potency, uptake, coverage and timeliness that influence the impact of immunization on the health and well-being of a given population.^3–6^ Many developing countries, including Nigeria, are making frantic efforts to ensure that life-saving vaccines are available for their citizens.^1^^,^^7^ These efforts to make quality vaccines available in the right quantity and conditions of storage at the last mile in the country is a collaborative effort between the government, at national and sub-national levels, and development partners such as the Vaccine Alliance (Gavi) and United Nations International Children’s Emergency Fund (UNICEF).^8–10^

Despite the efforts and commitments of developing countries to ensure vaccine coverage in their respective countries, some countries are still lagging in terms of immunization coverage, timeliness and completion rates.^5^^,^^6^^,^^11^ It was reported that every year, about 18.7 million infants are not reached with basic immunization services across the world^1^ and over 2 million children die from vaccine-preventable diseases every year.^12^ The timeliness of vaccine uptake is a very important factor that affects the success of any vaccination program and a point of concern for immunization programmers and policymakers.^13^ This is because the effectiveness of the administered vaccine is dependent on taking the complete regimen based on the standardized dosing intervals.

Immunization timeliness ensures that children are not exposed to infections before they get the protection available in vaccines and which could help to achieve some form of herd immunity.^13^^,^^14^ Another perspective on the public health importance of immunization timeliness is based on reports from different studies in developed and developing countries that children who are not vaccinated according to the schedule are much less likely to be fully vaccinated.^13–15^

A major gap in the approach of developing countries to ensure immunization coverage is their focus on improving the immunization supply.^3^ These efforts include the timely purchase and distribution of vaccines across the nooks and crannies of these countries. While this is commendable and a good development in many countries that before now did not demonstrate ownership of their vaccine programs, it is simply not enough to achieve the desired results necessary to ensure appropriate immunization coverage and timeliness.^16^

There is evidence in support of the need for programs promoting the demand side for immunization.^17^ This is because infants and children cannot decide whether they get vaccinated and when they should be vaccinated; thus, the significant role caregivers play in immunization uptake and timeliness cannot be overemphasized and the need to mobilize caregivers to improve immunization uptake, coverage and timeliness has been established in previous studies.^16–19^ No doubt, the bias and preference of caregivers affect their children when it comes to immunization; however, it has been reported that many caregivers simply fail to complete the immunization cycle for their children because they are unaware of the next immunization appointment or the number of required visits while other studies have reported that caregivers sometimes deprioritize immunization appointments.^20^^,^^21^

To effectively mobilize caregivers for immunization uptake and timeliness, some innovative approaches have been adopted. Some of these approaches leverage technology, for example, the use of digital registries and SMS reminders to caregivers but evidence of the impact of such interventions has been mixed and limited.^22^ These limitations are not farfetched considering the sociodemographic of a larger percentage of children that are not vaccinated or have not completed their cycle of vaccination. Many of the people in this category are not likely those with conditions such as access to the internet or mobile phones that will make mobile health (mHealth) interventions succeed.^23^^,^^24^ Some identified barriers reported in previous studies are lack of mobile phones,^22^^,^^24^ low literacy level among caregivers to read and comprehend messages,^25^ and frequent need to change phone numbers from what has been captured in the intervention databases.^25^^,^^26^

Despite the limitations of mHealth interventions to mobilize and remind caregivers of immunization schedules, the need to help caregivers know and remember immunization due dates still exists. As an adjunct to the conventional paper-based immunization cards that have been generally deployed across Nigeria, we designed and deployed a simple electronic, silicone-based wristband that flashes a light to remind caregivers of their immunization sessions. This innovation is called Wristband for Immunization Alert (WIA). This study, therefore, assessed the effectiveness and user-friendliness of WIA as a reminder device for caregivers to improve immunization uptake and reduce drop-outs.

## Methods

### Project design

**Fig. 1:**
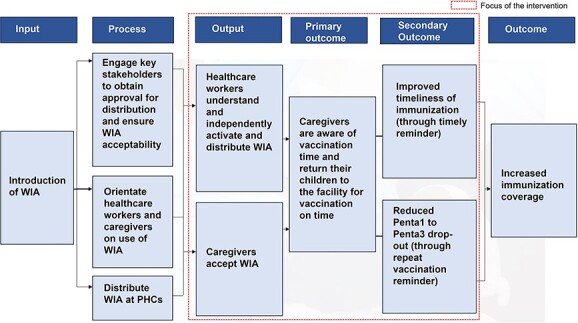
Process flow of WIA intervention.

A process flow of project implementation (Fig. 1) was developed in collaboration with relevant stakeholders in line with Nigeria’s goal of improving immunization coverage and data quality through electronic data capture and reminder systems. The intervention reviewed similar projects that have been implemented in Nigeria (Alma Sana band deployed in Nassarawa state, Vaccine Indicator Reminder band in Kebbi state and Bracelet for Immunization deployed in Bauchi state). Lessons from the project implementation as well as best practices informed the design and implementation of the project plan.

### The WIA device and its operation

**Fig. 2:**
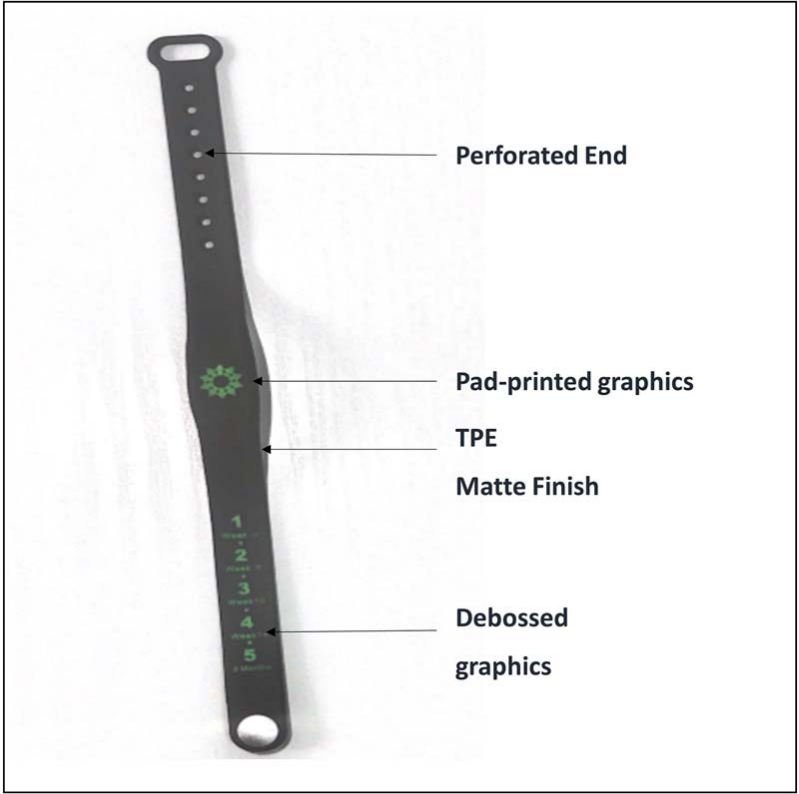
Picture of the WIA device showing the features.

**Fig. 3:**
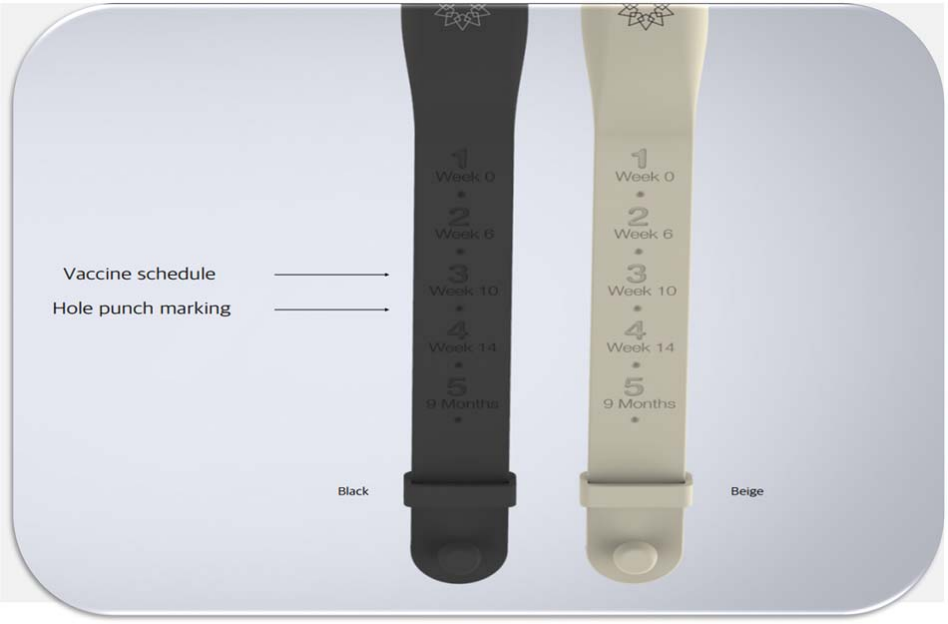
Picture of WIA showing the schedule of immunization.

**Fig. 4:**
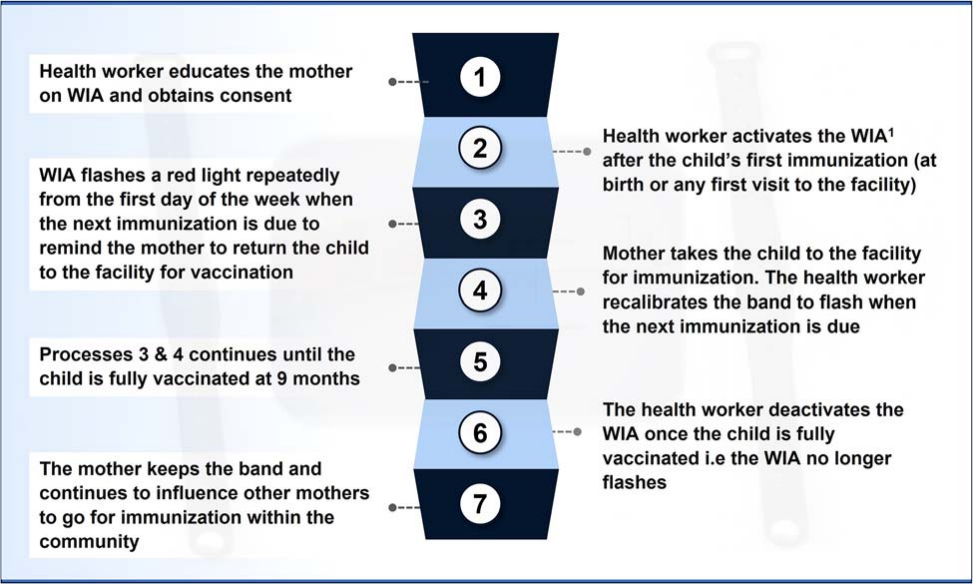
Operations of the WIA device.

WIA is an electronic wristband device made from an unreactive silicone material, and it has a lifespan of 12 months from the day it is activated. The features are shown in Fig. 2. WIA is worn by the caregiver and is programmed to flash a dim-red light before the child’s immunization to the day of the immunization (Fig. 3). The operation of the WIA device is shown in Fig. 4.

### Pilot testing

In line with the human-centered design approach, the WIA device was pretested on a smaller population of caregivers in one primary healthcare facility in a rural local government area (LGA) in Kano state. The result of the findings from field testing and their implications (relative to the approved concept) on the device and project designs are laid out in Table 1.

**Table 1:** Findings from the pilot of WIA and their implication

Findings from field testing	Caregivers’ rationale for the decisions or preferences in the findings	Implications on WIA and project design
The caregivers preferred that the wristband be worn by caregivers than the children	*Access:* The flashing of the red light can be easily spotted in the caregivers’ hands than the children’s who are often backed *Significance:* The wristband has a design synonymous with the ‘protective parental’ role of the mothers, hence, it resonates more with the mothers	The device was designed for the mothers to wear, as opposed to the initial target of the children

### Study design

This study adopted a two-arm approach that involved an intervention group and a control group. The intervention, which was the sole difference between the two groups, was the introduction of WIA that was given to the participants in the intervention group to serve as a reminder for their immunization dates appointment. Both groups had access to the full routine immunization services required.

#### Selection of primary healthcare centers for the study

This study was conducted in two local government areas (LGAs) in Kano state, Northwestern Nigeria. Gezawa and Minjibir LGAs were purposively selected based on their high rates of Penta drop-out and immunization left-out. We then selected four primary healthcare centers (PHCs) from each of the two selected LGAs based on the following criteria:

1. Only PHCs that offer immunization services.
2. PHC facilities with at least one staff who is responsible for recording routine immunization (RI) data.
3. In addition to the national and state ethical approvals obtained, PHCs whose officers-in-charge consented to the study.

The four PHCs from each LGA were drawn from both urban and rural communities. Thus, there were a total of four PHCs in the intervention group, and another four PHCs in the control group, with similar demographics. The control PHCs were completely blinded from the intervention, as neither the PHC staff nor caregivers were aware of the WIA intervention in the other PHCs.

*Exclusion*: Secondary, tertiary and privately owned health facilities.

#### Selection of caregivers

**Fig. 5:**
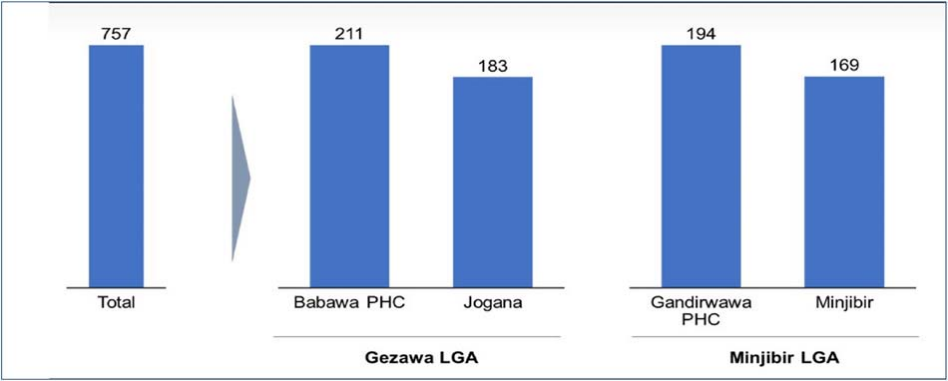
Distribution of caregivers for the WIA intervention from the two selected LGAs.

A total of 757 caregivers from the 4 intervention PHCs (Fig. 5) were selected for intervention because they met the following criteria:

1. Caregivers whose children were to receive routine immunization from the 1st to 4th visit
2. Caregivers with healthy children without complications at or after birth

*Exclusion*: Caregivers that declined participation, reported an intention to leave the community at any time before completing routine immunization, and those whose children were presenting for measles vaccine.

#### Immunization timelines

The approved immunization^27^ timelines that guided the implementation of this project is shown in Table 2.

**Table 2:** Approved immunization timeliness

		Interval between doses	
RI session	Age of 1st dose	Too early	Late
First (HBV)	0 week (at birth)	–	>2 weeks (age)
Second (Penta 1)	6 weeks	<6 weeks^a^	>6 weeks^a^
Third (Penta 2)	10 weeks	<4 weeks^a^	>8 weeks^a^
Fourth (Penta 3)	14 weeks	<4 weeks^a^	>8 weeks^a^
Fifth (Measles 1)	9 months	<24 weeks (age)	>40 weeks (age)

a Interval between multiple vaccination doses.

#### Data collection and analysis

To assess the effectiveness of WIA as a reminder device for caregivers to improve immunization uptake, data collection tools were designed on Microsoft Excel and adapted from the facility register template to capture the dates that caregivers take their wards for their first immunization and return for subsequent doses up until the fifth dose for measles vaccination. The baseline immunization timeliness data for the intervention group were collected from the selected health facilities for one calendar year before the intervention dates. At the end of the intervention, immunization timeliness data for the period of intervention (August 2019 to March 2020) were mined from the PHC facility registers of the control and intervention PHCs. These were then analyzed using Microsoft Excel and IBM SPSS version 21. A difference-in-difference analysis was conducted to compare the baseline and end-line results.

#### Assessment of the user-friendliness of WIA

To assess the user-friendliness of WIA as a reminder device for caregivers, 12 key informant interviews (KIIs) were conducted with health workers at the pilot facilities to understand the purpose of the wristband and assess the ease of use by health workers. A total of 51 KIIs were also conducted with the caregivers that received the WIA device to assess the usefulness of the wristband as a reminder tool, and its acceptability by the caregivers. Only the health workers and caregivers who consented to participate were interviewed.

The interviews with the health workers and caregivers were conducted in Hausa, being the local language of the community. The interviews were first translated into English language, then transcribed. The transcripts were read for a general overview, clarity and comprehension. A thematic framework was then developed from the narratives of the discussants. Descriptive statements were formed, and quotes were lifted from their original statements to explain the identified themes.

To further assess WIA quality and collate feedback arising from its use, a feedback form was provided at the intervention PHCs to document complaints, allergic reactions, malfunctioning, etc. Upon the presentation of the caregivers at the PHCs for their ward’s vaccination, the health worker asks for complaints or feedback from the caregiver and documents and then vaccinates the ward. The feedback forms were collated and analyzed at the end of the intervention.

*Ethical considerations*: Informed consent was obtained from all participants in the study and participants were duly informed of their rights to withdraw participation from the study at any point in the study. All data obtained were treated confidentially and used for research and advocacy purposes only. The study adhered strictly to the cardinal principles of beneficence and non-maleficence in the conduct of research. Ethical approval was obtained from the Kano state primary health care board and the Federal Capital Territory Health Research Ethics Committee (FHREC) with ref number: FHREC/2020/01/139/21-12-2020.

## Results

The main outcome of the study was to measure the timeliness in reporting for immunization and reduction in drop-out rates, and this was done at the completion of the intervention, which lasted 9 months.

### Effectiveness of WIA as a reminder device: immunization timeliness

**Fig. 6:**
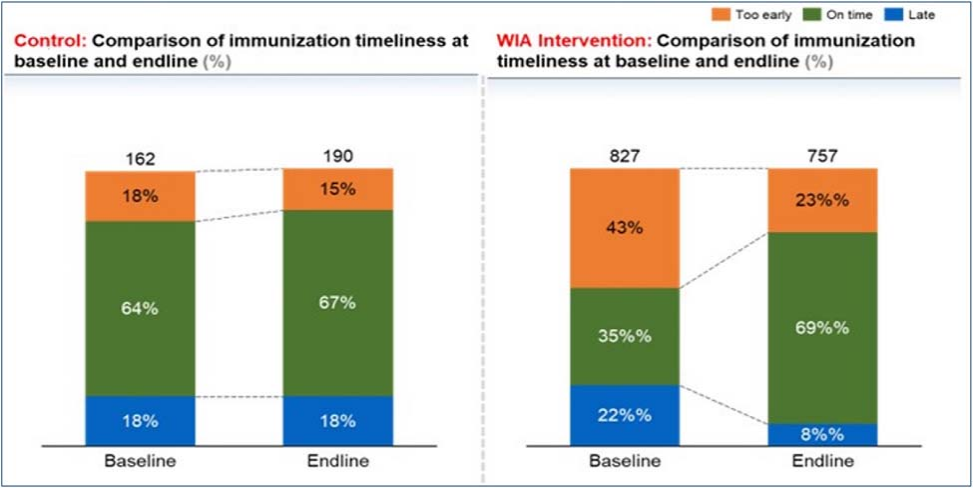
Comparison of the baseline and end-line immunization timeliness between the control and intervention groups.

**Fig. 7:**
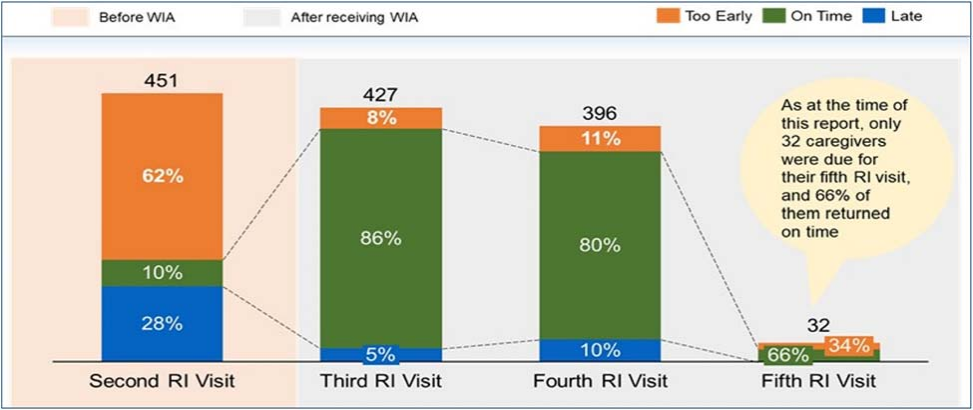
Cohort analysis of caregivers who got WIA in their second visit in the pilot PHCs (%).

The comparison of the baseline and end-line immunization timeliness between the control group and intervention showed a 34% improvement in the RI timeliness from 35% at the baseline to 69% at the end-line for the intervention group while only a 4% increase was recorded for the control (Fig. 6). Also, the number of caregivers who reported early for immunization in the intervention group decreased by 20% while those that reported late decreased by 14%, from baseline to end-line, compared to the control (Fig. 6). An upward trend of over 80% routine immunization timeliness was observed among cohorts of caregivers who received WIA in their first and second visits. Only 32 caregivers were due for their fifth RI visit at the end of the pilot phase of the WIA intervention; however, 66% of them returned on time (Fig. 7).

### Effectiveness of WIA as a reminder device: routine immunization drop-out rate

**Fig. 8:**
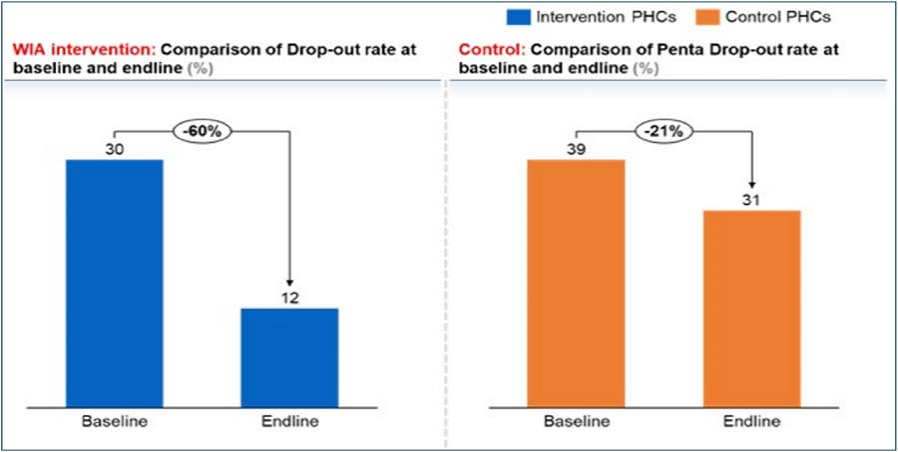
Drop-out rates in the intervention and control groups.

A comparison of the drop-out rates during the baseline and end-line for the intervention group showed a 60% reduction in immunization drop-out rates while a 21% drop-out rate was recorded for the control group (Fig. 8).

### A difference-in-difference analysis of the effect of WIA intervention on immunization timeliness

The difference-in-difference analysis between the control and intervention groups indicated a 30% increase in immunization timeliness between the baseline and end-line associated with WIA introduction (Table 3).

**Table 3:** Difference-in-difference analysis between control and intervention groups showing the difference in immunization timeliness between the baseline and end-line

	Baseline	End-line	Difference (baseline–end-line)	Difference in difference
Control	63%	67%	4%	
Intervention	35%	69%	34%	
Difference (control—intervention)	28%	−2%		30%
Difference in difference			30%	

### Relative risk analysis

The relative risk of caregivers returning for vaccination of their child on time, based on the RI schedule as shown in Table 4, is identical, i.e. close to 1, across the age groups of women in the intervention cohort except for women 35 years and above with a relative risk of 0.4786, which increased in subsequent visits.

**Table 4:** Relative risk (RR) of vaccination timeliness among caregivers in the intervention group

Caregiver’s age (years)	Relative risk		
	Timeliness @2nd visit	Timeliness @3rd visit	Timeliness @4th visit
<20	0.8341	1.0041	1.099
20–24	1.2222	0.9873	0.9978
25–29	0.7859	0.9899	0.9088
30–34	1.3788	1.0377	1.0416
>35	0.4786	0.9780	1.0323

### User-friendliness of the WIA device: ease of use by healthcare workers

Findings from the key informant interviews showed that all the health workers in charge of routine immunization and the WIA field assistants demonstrated a full understanding of the purpose of the wristband and how to reset the wristband when the caregivers return for subsequent vaccinations.

### User-friendliness of WIA device: acceptability by caregivers

Findings from the interviews showed that all the caregivers (100%) engaged at the pilot facilities demonstrated a good understanding of the purpose of the wristband, as all the respondents could articulate in their own words how the wristband functions and their expectations of it to signal when immunization is due. There was also no occurrence of discontinuation of use of the WIA device after the first visit hence depicting a 100% acceptance of the wristbands by the caregivers.

### Feedback on WIA quality

The result analyzed for feedback on WIA showed that there was no complaint of its malfunction; however, the caregivers largely reported friction (19.9%), rubbing (86.1%) and skin irritation (9.4%) around the wrist where the WIA device was worn. No other allergic reaction was documented.

## Discussion

### Main findings of this study

Our study captured results of immunization timeliness up to the 5th visit of immunization, and it is an improvement over the result of a similar intervention,^3^ which only captured immunization timeliness up to the 2nd visit after the intervention. While there was a 4% increase in immunization timeliness in the control group, the intervention group that recorded a 34% improvement could be attributed to the introduction of WIA. The WIA device helped to improve immunization timeliness and recorded a decrease in the number of caregivers who reported early for immunization. Decreasing the rate at which caregivers report early for immunization is expected to help improve timeliness. Our study also showed that drop-out rates decreased by 60%. It is pertinent to note that the result of our drop-out rate was captured at the end of the intervention. This means that there is still the possibility of the caregivers that were recorded to have dropped out to return for immunization. The difference-in-difference analysis that compared the difference between the difference in immunization timeliness in both the control and intervention groups showed a 30% improvement in immunization timeliness at the end-line. This improvement can be attributed to the WIA device as a reminder to the caregivers. WIA can be categorized alongside the ‘Reminder/recall’ interventions that have been deployed across developing countries including Nigeria.^28^

The result of the relative risk analysis of the caregivers returning timely for the 2nd, 3rd, and 4th immunization was identical for the first four age groups (between 20), which implies that the caregivers would most likely return early for immunization. The relative risk of caregivers in the last age group (above 35 years) returning for vaccination during the 2nd visit is low. However, it is high for subsequent visits. The caregivers in this group may have found it difficult to adjust to the prompting of the WIA device during the 2nd visit but got used to it in subsequent visits. In addition, the study of Hu *et al.* on the completeness and timeliness of vaccination and determinants for low and late uptake among young children in eastern China showed that mother’s age was a predictor of vaccination timeliness as younger mothers had increased immunization rates and higher probability of timeliness of vaccination because they have a better antenatal and medical care utilization.^29^

The user-friendliness of the WIA device was shown in the understanding of the functions and in the ability of the health workers to reset the device for subsequent vaccinations. The study identified the health workers as the major stakeholder; hence, they are responsible for resetting the device against the next immunization. This means that their role in promoting or discouraging the use of such health innovations cannot be overemphasized. Findings have aligned with our study, highlighting the role of health workers in promoting health innovations.^30^^,^^31^ There is then a need for health innovations to be designed in such a way that health workers and receivers can use them with ease. This is because a misunderstanding of the use of the WIA device on the part of the health workers would have defeated the purpose of the intervention.

The caregivers captured in this study also demonstrated a good understanding of the purpose of the device. This is a testament to its acceptability and expectations of WIA as a reminder device. The caregivers who are the key drivers of immunization timeliness reported WIA to be helpful and expressed a desire to recommend it to others. For this kind of technological innovation to be successful, caregivers must be willing to use the device. This is because they are responsible for taking their children/wards to the immunization center. In a similar study conducted in Sokoto, another Northwestern state in Nigeria, vaccination coverage was said to be influenced by two caregiver-dependent factors: caregivers skipping immunization due to the pain to the child and not knowing when to come for vaccination.^32^ Another similar study conducted in eight Northern states in Nigeria reported caregivers’ lack of immunization timeliness (50%) and forgetting immunization schedule (16%) as reasons for not receiving all vaccinations.^33^ Therefore, irrespective of the intervention, caregivers/users must express willingness and desire to use technological innovations/interventions for them to be successful.^32^^,^^33^ The acceptance and continued use of the WIA device by the caregivers in this study is an important validation of the wristband in the context of value reorientation, especially in the Northwestern part of Nigeria where religious and cultural beliefs tend to checkmate technological innovations.

Although there were reports of friction due to the tightening of the strap, it was resolved by reducing the tightening. Most of the caregivers reported rubbing and skin irritation due to long-term use of the WIA device. It is widely known that a tight band/strap causes skin irritation while a loose strap/band causes rubbing.^34^^,^^35^ It was also documented that caregivers were aware of this, and it did not result in drawbacks.

### What is already known on this topic

A mixed result of other interventions like short message service (SMS), postal and telephone reminders, incentivizing caregivers, door-to-door visits, as well as community-based counseling have been shown to vary from one community to the other, toward improving immunization timeliness.^36^ Furthermore, the effectiveness of SMS is linked to the educational level of the caregiver, as well as the availability and accessibility of mobile phones and network issues.^37–39^ In addition, door-to-door visits which are expensive and not sustainable as well as a lack of human resources and high costs/lack of immunization records have been reported to pose challenges to the acceptance of more technology-based reminder services.^40^ Therefore, our study, which showed improvement in immunization timeliness, has the potential for addressing the pervasive challenges that have been reportedly attributed to immunization timeliness.

### What this study adds

In a setting with poor immunization timeliness and drop-out rates, the WIA project intervention can significantly improve immunization timeliness and reduce drop-out rates. Given that immunization timeliness and drop-outs are reported issues of concern in Northwestern Nigeria, the use of WIA devices has been shown to be a solution. While there has been a different approach to improving the timeliness of immunization and reducing drop-out rates, especially in the Northern parts of Nigeria (Alma Sana band in Nassarawa state, Vaccine Indicator Reminder band in Kebbi state and Bracelet for Immunization in Bauchi state), none of these interventions could compare to the uniqueness of WIA. This is because WIA is electronic, water resistant, easy to use and worn by caregivers as opposed to the other innovations (bracelets and wristbands) that are worn on the ankle or wrists of the children. In a similar study in Pakistan, caregivers complained of the inappropriate size of the bracelet for their children^3^; hence, they tend to wear the bracelets only when they were reporting for immunization. This means that WIA will be more appropriate in rural settings where illiterate caregivers may not be able to read SMS alerts as reminders. Caregivers will also find it easier to wear the WIA device on their wrists, compared to having such a device on the wrist or ankles of their children.

### Limitations of this study

This study area has moderate immunization levels, which are often influenced by religious beliefs. Also, the educational level of these caregivers might have contributed to poor immunization timeliness and drop-out rates. Thus, this study is not recommended in areas where caregivers are well informed about the advantages of immunization. A major limitation of our study was that it did not measure how the educational level of the caregivers may have impacted the study, given that other studies of similar interventions attributed differences in their results to the level of education of the caregivers. Also, no bias was introduced to the study as the control PHCs were not visited until after the WIA intervention had been completed. However, the possibility of the healthcare workers and caregivers at the control PHCs being aware of the WIA device could not be completely guaranteed, as the PHCs in the control and intervention groups were located in the same LGA, even though they were a distance apart, and in different communities.

## Conclusion

The WIA device aided the timeliness of immunization and reduced the immunization drop-out rate among the intervention group compared with the control group. Given that immunization timeliness and drop-outs are reported issues of concern in Northwestern Nigeria, the use of the WIA device that has been shown by this study as a solution is a recommended intervention.

### Recommendations

There are other factors such as challenges with logistics on the part of the caregivers that could have also influenced the timeliness of immunization. These other factors were not explored in this study; hence, we recommend that further studies be conducted to explore these factors and their effects on immunization timeliness.

## Data Availability

The datasets used and/or analyzed during this study are available from the corresponding author (oluwafisayo.ayodeji@sydani.org, oluwafisayorichie@gmail.com) upon reasonable request

## List of Acronyms

LGA–Local government area; PHC–Primary healthcare centre; RI–Routine immunization

## Notes

**Sidney Sampson**, Managing Partner, CEO (MPH)

**Adebisi Adenipekun**, Engagement Manager (MPH)

**Sunday Atobatele**, Principal (M.Sc)

**Oluwafisayo Ayodeji**, Research Associate (PhD)

**Oluomachukwu Omeje**, Associate (MPH)

**Folake Oni**, Principal (MBBS, MBA)
